# A New Monoterpene from the Leaves of a Radiation Mutant Cultivar of *Perilla frutescens* var. *crispa* with Inhibitory Activity on LPS-Induced NO Production

**DOI:** 10.3390/molecules22091471

**Published:** 2017-09-04

**Authors:** Bomi Nam, Yangkang So, Hyo-Young Kim, Jin-Baek Kim, Chang Hyun Jin, Ah-Reum Han

**Affiliations:** Advanced Radiation Technology Institute, Korea Atomic Energy Research Institute, Jeongeup-si, Jeollabuk-do 56212, Korea; bomi1201@kaeri.re.kr (B.N.); yangkang@kaeri.re.kr (Y.S.); khy5012@kaeri.re.kr (H.-Y.K.); jbkim74@kaeri.re.kr (J.-B.K.); chjin@kaeri.re.kr (C.H.J.)

**Keywords:** *Perilla frutescens* var. *crispa*, radiation mutant cultivar, 9-Hydroxy-isoegomaketone, anti-inflammation

## Abstract

The leaves of *Perilla frutescens* var. *crispa* (Lamiaceae)—known as ‘Jureum-soyeop’ or ‘Cha-jo-ki’ in Korean, ‘ZI SU YE’ in Chinese, and ‘Shiso’ in Japan—has been used as a medicinal herb. Recent gamma irradiated mutation breeding on *P. frutescens* var. *crispa* in our research group resulted in the development of a new perilla cultivar, *P. frutescens* var. *crispa* (cv. Antisperill; PFCA), which has a higher content of isoegomaketone. The leaves of PFCA were extracted by supercritical carbon dioxide (SC-CO_2_) extraction, and phytochemical investigation on this extract led to the isolation and identification of a new compound, 9-hydroxy-isoegomaketone [(2*E*)-1-(3-furanyl)-4-hydroxy-4-methyl-2-penten-1-one; **1**]. Compound **1** exhibited inhibitory activity on nitric oxide (NO) production in lipopolysaccharide (LPS)-activated RAW264.7 cells with an IC_50_ value of 14.4 μM. The compounds in the SC-CO_2_ extracts of the radiation mutant cultivar and the original plant were quantified by high-performance liquid chromatography with diode array detection.

## 1. Introduction

Perillae Folium is the dried leaves of *Perilla* species (Lamiaceae) including *P. frutescens* var. *frutescens*, *P. frutescens* var. *crispa*, and *P. frutescens* var. *acuta*. Perillae Folium has a pungent taste and a warm property acting on the lung and spleen channels, and it has been also used as a diuretic, sedative, detoxifying, and antipyretic agent in the traditional medicine of East Asia [1]. The leaves of *Perilla frutescens* var. *crispa* (Lamiaceae) have been ethnopharmacologically used to protect the digestive tract from inflammatory diseases and to prevent sea-food poisoning [2]. Monoterpenes in the essential oil of *P. frutescens* var. *crispa* are the major components [3], with diverse biological effects including antifungal [4], neuroprotective [5], anticancer [6,7], angiogenesis inhibitory [8], anti-inflammatory [9], and antioxidant activities [10]. In addition, various types of anthocyanin [11], phenolics [12,13], polysaccharides [14], and flavonoids [15] have also been isolated from this plant. Some of these compounds were found to have diverse biological activities such as antioxidant [12], immune adjuvant [14], anti-inflammatory [15,16], and antioxidant effects [17].

**Figure 1:**
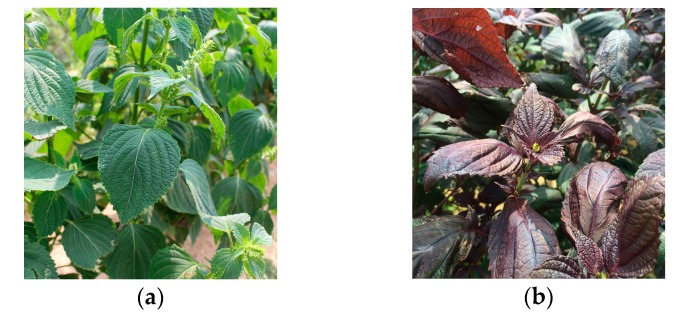
*Perilla frutescens* var. *crispa* (Lamiaceae): (**a**) A mutant cultivar, *P. frutescens* var. *crispa* (cv. Antisperill; PFCA), which developed by 200 Gy gamma irradiation from a labeled Cobalt (^60^Co) source on seeds of an original cultivar; (**b**) an original cultivar (PFC) that has red-purple leaves.

**Figure 2:**
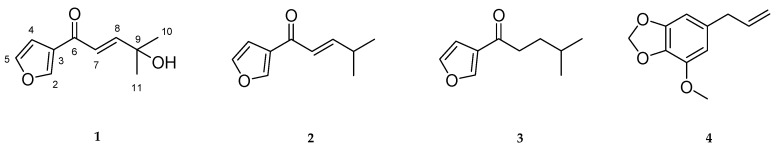
Chemical structures of compounds from the SC-CO_2_ extracts of PFCA and PFC.

Mutation breeding generated by ionizing generation has been used to improve crop productivity and quality for many years and has led to more than 3000 mutant varieties of plants [18]. Our research group has developed a gamma irradiated mutant cultivar of *P. frutescens* var. *crispa* (cv. Antisperill; PFCA), with a high content of isoegomaketone (**2**) compared to the original cultivar [13] (Figure 1). In our previous biological evaluations on **2**, this compound was found to have anticancer [7], anti-inflammatory [9], and antioxidant activities [10]. Recently, we reported that the PFCA extract by supercritical carbon dioxide (SC-CO_2_) extraction method contained the increased content of monoterpenes and enhanced anti-inflammatory effect, compared to those of the SC-CO_2_ extract of the original cultivar (PFC) [19] as well as the ethanol extract of PFCA [20]. SC-CO_2_ extraction technology has been recognized as a powerful technique for essential oils from oil bearing plant sources [21], with advantages of being non-toxic, non-flammable, time and cost-effective, and more environmentally friendly [22]. Other than **2** and perilla ketone (**3**), two unknown compounds were found in the SC-CO_2_ extracts of PFCA and PFC by HPLC analysis [19,20]. Therefore, repeated column chromatography of the SC-CO_2_ extracts of PFCA and PFC led to the isolation of a new compound **1** and myristicin (**4**) [23], respectively (Figure 2). The structure elucidation and biological evaluation of **1** and quantitative analyses of the components of PFCA and PFC are described herein.

## 2. Results

### 2.1. Structure Elucidation of Compound ***1***

**Figure 3:**
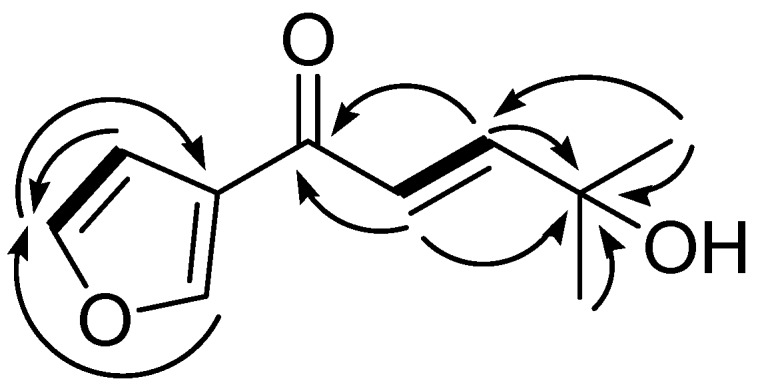
Key ^1^H-^1^H COSY (▬) and ^1^H-^13^C HMBC (→) correlations of **1**.

Compound **1** was obtained as a colorless oil, with a molecular ion peak [M]^−^ at *m*/*z* 180.0788 in the HRESIMS spectrum, corresponding to an elemental formula of C_10_H_12_O_3_. The UV absorption maxima at 230 and 254 nm indicated the presence of aromatic ring and conjugated enone group [24]. The ^1^H- and ^13^C-NMR spectra of **1** were similar to those of isoegomaketon [25], except for the absence of signals for a methine group and the presence of an oxygenated tertiary carbon signal at δ_C_ 71.3 (C-9). The ^1^H- and ^13^C-NMR spectra of **1** displayed signals for an aromatic system at δ_H_ 7.46 (1H, s, H-2)/δ_C_ 144.4 (C-2), 6.84 (1H, s, H-3)/109.1 (C-3), and 8.81 (1H, s, H-5)/147.6 (C-5), and a conjugated carbonyl carbon at δ_C_ 184.6 (C-6), suggesting that **1** has a 3-furylketone, supported by the ^1^H-^13^C HMBC correlations of H-2/C-4, C-5, H-4/C-2, C-5, and H-5/C-2, C-3, C-4 (Figure 3). The ^1^H NMR signals for two uncoupled methyl groups at δ_H_ 1.40 (6H, s, H-10 and H-11), a *trans*-olefinic group at 6.77 (1H, d, *J* = 15.3 Hz, H-7) and 7.08 (1H, d, *J* = 15.3 Hz, H-8), and the ^13^C-NMR signal for an oxygenated tertiary carbon at δ_C_ 71.3 (C-9) showed the presence of an isoprenyl alcohol group, positioned at the C-6 as evidenced by the ^1^H-^13^C HMBC correlations of H-7/C-6, C-9 and H-8/C-6, C-9 (Figure 3). Further detailed analysis of ^1^H-^1^H COSY, ^1^H-^13^C HSQC, and ^1^H-^13^C HMBC NMR data allowed unambiguous assignments for all of the ^1^H- and ^13^C-NMR signals of **1** (Figures S1−S5). Therefore, the structure of **1** was determined as (2*E*)-1-(3-furanyl)-4-hydroxy-4-methyl-2-penten-1-one and this compound was named as 9-hydroxy-isoegomaketone.

### 2.2. Effect of Compound ***1*** on NO production in LPS-Activatied RAW 264.7 Cells

**Figure 4:**
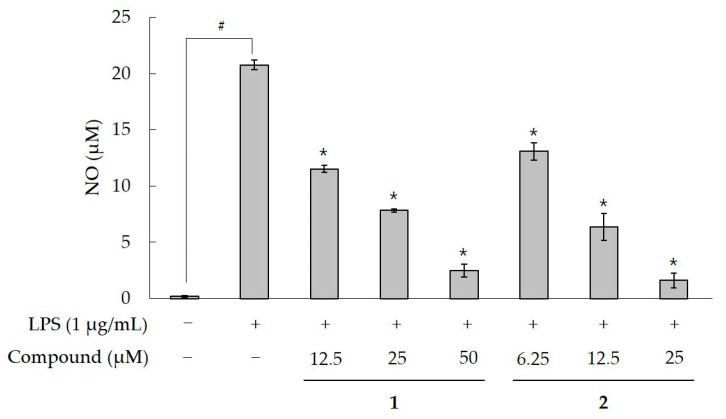
Effects of **1** and **2** on NO production in RAW 264.7 cells. Data are presented as means ± SD (*n* = 6). ^#^ *p* < 0.01 vs. negative control. * *p* < 0.05 vs. the LPS-alone group.

NO is a gaseous free radical produced by the oxidation of l-arginine catalyzed by NO synthases (NOS), with a wide range of physiological and pathological actions. Among the isoforms of NOS—neural (nNOS), endotherial (eNOS), and inducible (iNOS)—iNOS is induced in response to bacterial LPS and proinflammatory cytokines. Therefore, the inhibition of LPS-induced NO production is considered as an inhibitor of iNOS, which is a mediator of inflammation and carcinogenesis [26]. Compound **1** decreased the LPS-stimulated NO production in a concentration-dependent manner (Figure 4) and showed inhibitory activity with an IC_50_ value of 14.4 μM. However, it inhibited the LPS-induced NO production less effectively than **2** (IC_50_, 8.8 μM). Cytotoxicities of compounds were also determined by using the EZ-Cytox cell viability assay kit. Compound **1** did not affect cell viability at any of the tested concentrations, but **2** was found too toxic at the concentrations ≥25 μM (data not shown) [8].

### 2.3. Quantitative Analysis of Four Compounds from Two Perilla Cultivars

**Figure 5:**
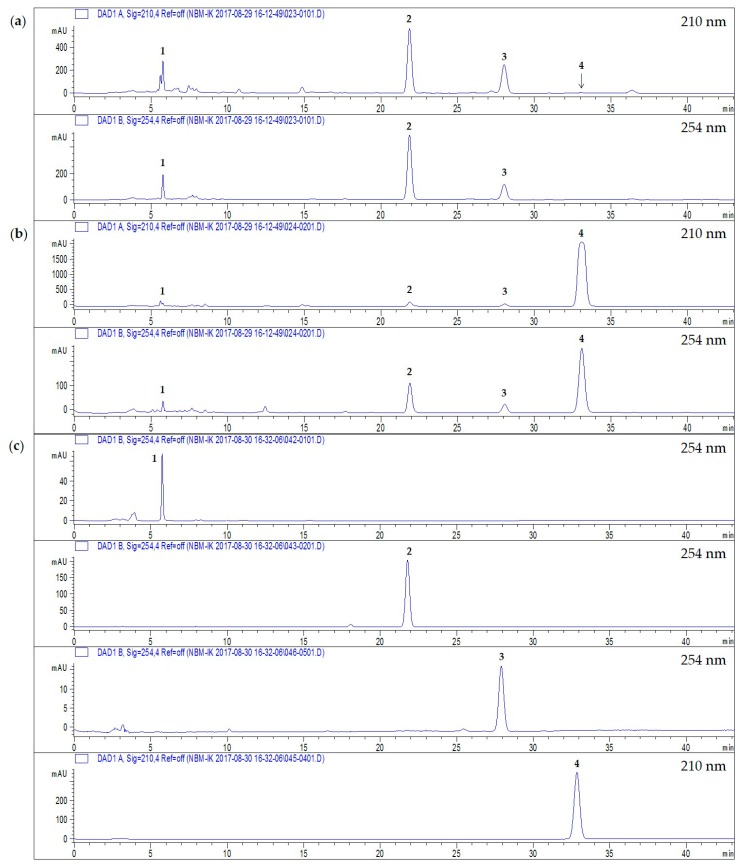
HPLC chromatograms of (**a**) the SC-CO_2_ extract of PFCA at 210 and 254 nm; (**b**) the SC-CO_2_ extract of PFC at 210 and 254 nm; (**c**) the four standards: 9-hydroxy-isoegomaketone (**1**), isoegomaketone (**2**), and perilla ketone (**3**) at 254 nm and myristicin (**4**) at 210 nm.

The HPLC-DAD analytical method established in our previous studies [19,27] was applied to the simultaneous determination of four compounds in the SC-CO_2_ extracts of PFCA and PFC (Figure 5). The four components were separated on a reverse phase analytical column using a gradient solvent system of acetonitrile and water. The ultraviolet wavelength used for detection was 254 nm. The linearity of this analytical method was evaluated based on the correlation coefficient (*r*^2^) value of the calibration curves of each compound. The calibration curves of the four compounds were obtained by the assessment of the peak areas of the standard solutions at four different concentrations. The calibration curves showed a high degree of linearity with an *r*^2^ > 0.9991 over the concentration ranges 20−100 μg/mL for **1** and **3**, 20−164 μg/mL for **2**, and 100−1000 μg/mL for **4** (Table 1). The limits of detection (LOD) and limits of quantitation (LOQ) for the four components were calculated using the slope of the calibration curve and the standard deviation (SD) of the intercept. The LODs and LOQs for the compounds **1**–**4** were in the range 0.006−0.124 μg/mL and 0.116−0.952 μg/mL, respectively. The amounts of the four compounds are listed in Table 2. The contents of **1**–**3** were approximately 9-, 5-, and 4-fold higher, respectively, in the SC-CO_2_ extracts of PFCA than in those of PFC. Compound **4** was not observed in the SC-CO_2_ extracts of PFCA; however, it was the most abundant in the SC-CO_2_ extracts of PFC.

## 3. Discussion

**Figure 6:**
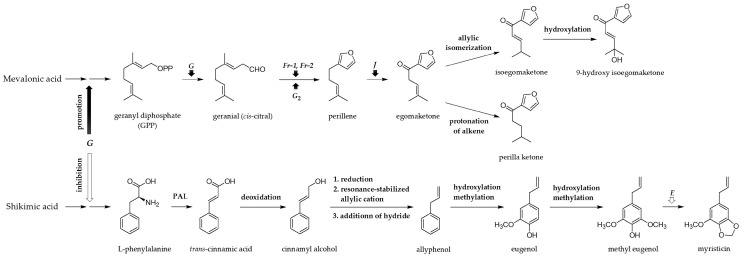
Proposed biosynthetic pathway for four components found in PFCA and PFC [28,29,30,31,32].

In the comparison studies of LPS-induced anti-inflammatory activities and chemical profiles of the SC-CO_2_ extract of PFCA versus PFC [19] and the SC-CO_2_ versus the ethanol extracts of PFCA [20], the SC-CO_2_ extract of PFCA showed greater potency and higher content of **2** and **3** than the SC-CO_2_ extract of PFC and the ethanol extract of PFCA. The HPLC chromatogram of these three extracts showed an unknown peak besides the two main peaks for **2** and **3**, and thus it was identified as compound **1**. While, another unknown peak, which appeared in the chromatogram of the SC-CO_2_ extract of PFC, but not in the chromatograms of the SC-CO_2_ and the ethanol extracts of PFCA, was identified as compound **4**. Therefore, the accumulation of **1**–**3** and the degradation of **4** in PFCA leaves are thought to be associated with the variation in the metabolic pathways by gamma irradiation effect. The biosynthesis of new compound **1** can be inferred from several studies on the biosynthetic pathway of monoterpene and phenylpropanoid proposed from their genetic analysis in *P*. *frutescens* (Figure 6) [28,29,30,31]. Compounds **2** and **3** were synthesized from a precursor, geranyl diphosphate (GPP) through the mevalonate pathway. In this reaction, GPP gave rise to a linear monoterpene, geranial (*cis*-citral) by promotion of dominant gene *G* which is considered to be essential for the initiation of monoterpene biosynthesis [29]. Geranial (*cis*-citral) was converted to perillene through the furan formation controlled by the polymeric genes (*Fr1* and *Fr2*) [30] and (or) the oxidation of *cis*-citral controlled by gene *G*_2_, and then the conversion of perillene into egomaketone was promoted by the dominant gene (*J*) that controlled the oxidation of C-6 position of perillene [31]. Based on the construction mechanisms [32], **2** and **3** would be expected to be synthesized by the allylic isomerization and the protonation of alkene, respectively, of the precursor egomaketone. Further hydroxylation of **2** was assumed to create 9-hydroxy-isoegomaketone (**1**) of a new structure type (Figure 6). While, in the absence of gene *G*, phenylpropanoids accumulated instead of monoterpenes in the perilla plants [28]. Compound **4** was known to be produced in the biosynthesis via the shikimate pathway [32]. Shikimic acid has a role in the formation of aromatic acids, l-phenylalanine, l-tyrosine, and l-tryptophan. Among them, l-phenylalanine generates *trans*-cinnamic acid by the elimination of ammonia from its side-chain via phenylalanine ammonia lyase (PAL) which is an enzyme to catalyze the reaction converting l-phenylalanine to *trans*-cinnamic acid. Deoxidation from *trans*-cinnamic acid generates cinnamyl alcohol, and then eugenol is derived from cinnamyl alcohol by the following steps: loss of hydroxyl as leaving group, resonance form of the allylic cation, and addition of hydride [32]. Tabata (2000) suggested that a possible precursor—methyleugenol—was metabolized to compound **4** by the formation of a methylenedioxy group, in the absence of a dominant gene *E* which is involved in the production of elemicin. However, to understand the mechanisms responsible for the accumulation of monoterpenes in the gamma-irradiated mutant cultivar PFCA, further enzymological and gene function studies are still necessary.

Medicinal plants have historically been valuable sources of therapeutic agents, and still an interest in the discovery of novel drug leads from natural products is on the increase [33,34,35]. In continuation of our search to find new active compounds from plants and their improved varieties (cultivars), a new compound **1** was isolated from a new cultivar—PFCA—and showed the inhibitory activity in the measurement of NO production on LPS-stimulated macrophage cells. Compound **2** showed greater inhibitory activity with an IC_50_ value of 8.8 μM than that of **1** (IC_50_, 14.4 μM), but its inhibitory activity on NO production seems to be related to its concomitant cytotoxic effect. Thus, Compound **1** may be considered as an active and safe component of PFCA for targeting inflammation disease. Further mechanistic studies using in vitro and in vivo models of inflammation disease will be performed to verify the anti-inflammatory activity of **1**.

## 4. Materials and Methods

### 4.1. General Procedures

The NMR experiments were performed using a JNM-ECA 500 MHz NMR instrument (JEOL Ltd., Tokyo, Japan). HRESIMS was carried out on a JMS-700 MStation Mass Spectrometer (JEOL Ltd., Tokyo, Japan). UV spectra were recorded on an Evolution 260 Bio UV–Visible spectrophotometer (Thermo Fischer Scientific, Waltham, MA, USA). Thin-layer chromatographic (TLC) analysis was performed on Kieselgel 60 F254 (Merck, Darmstadt, Germany) and Kieselgel 60 RP-18 F254S (Merck), with visualization performed under UV light (254 and 365 nm) and 10% (*v*/*v*) sulfuric acid spray followed by heating (200 °C, 2 min). YMC Gel ODS-A (12 nm, S-150 μm; YMC Co., Kyoto, Japan) was used for column chromatography (CC). Preparative HPLC was performed using a Gilson Preparative HPLC system (Gilson Inc., Middleton, WI, USA) equipped with YMC Pack Pro C18 (5 μm, 250 × 20 mm, YMC Co.). Analytical HPLC was performed using an Agilent 1100 series system (Agilent Technologies, Palo Alto, CA, USA) equipped with YMC-Triart C18 (5 μm, 250 × 4.6 mm, YMC Co.). A [^60^Co] γ-irradiator (150 TBq capacity; AECL, Ottawa, Canada) was used for gamma irradiation. All other chemicals and solvents used in this study were of analytical grade.

### 4.2. Plant Materials

*Perilla frutescens* var. *crispa* (cv. Antisperill; PFCA) is a mutant perilla cultivar that produces green leaf with a high content of isoegomaketone (**2**). It was developed by 200 Gy gamma irradiation from a labeled Cobalt (^60^Co) source on seeds of the original plant, *Perilla frutescens* var. *crispa*, with a red-purple leaf and examined as stable inheritance of phenotype for three years (1995–1998) at the Advanced Radiation Technology Institute, Korea Atomic Energy Research Institute (Jeongeup-si, Jeollabuk-do, Korea). PFCA leaves were collected each year shortly before the flowering time. PFCA seeds have been deposited for the purpose of patent procedures in the Korean Collection for Type Cultures, Biological Resource Center, Korea Research Institute of Bioscience and Biotechnology (August, 2016). The voucher specimens have been deposited at the Advanced Radiation Technology Institute, Korea Atomic Energy Research Institute (Jeongeup-si, Jeollabuk-do, Korea).

### 4.3. Extraction and Isolation

The dried leaves of PFCA (300 g) were pulverized and then extracted by SC-CO_2_ extraction method using a laboratory-scale supercritical fluid extraction system (Ilshin Autoclave Co., Daejeon, Korea). The powdered sample was placed into the extraction column of SC-CO_2_ extractor. The predetermined conditions are as follows: pressure, 400 bar; temperature, 40 °C. The flow rate of CO_2_ (99.9%) was constant at 60 mL/min, for an extraction time of 3 h. The oil was collected 13.81 g (4.6% *w*/*w*) and stored in a refrigerator at 4 °C. A part (2 g) of the extract was subjected to RP-C_18_ CC (MeOH–water, 1:1 to 9:1, *v*/*v*) to yield 11 fractions (F01–F11). Fraction F03 (25 mg) was chromatographed on sephadex LH-20 using 100% MeOH to give five sub-fractions (F0301-F0305). The third fraction (F0303, 11 mg) was purified by preparative HPLC (YMC Triart C18, MeOH–water = 2:3, 4 mL/min, UV 280 nm) to yield **1** (*t*_R_ 21.03 min, 6 mg). The dried leaves of PFC (300 g) were extracted by the SC-CO_2_ extraction method under the same conditions as mentioned above, affording 14.84 g of oil extract (4.9% *w*/*w*) was obtained. A part (1 g) of the extract was subjected to RP-C_18_ CC (MeOH–water, 2:1 to 4:1, *v*/*v*) to yield twenty fractions (F01–F20) and pure compounds **2** (2 mg), **3** (5 mg), and **4** (9 mg).

9-Hydroxy-isoegomaketone ((2*E*)-1-(3-furanyl)-4-hydroxy-4-methyl-2-penten-1-one; **1**). Colorless oil. UV (MeOH) *λ*_max_ (log ε) 230 (3.69), 254 (3.44) nm; ^1^H-NMR (CDCl_3_, 500 MHz) δ 8.81 (1H, br s, H-5), 7.46 (1H, s, H-2), 7.08 (1H, d, *J* = 15.3 Hz, H-8), 6.84 (1H, br s, H-4), 6.77 (1H, d, *J* = 15.3 Hz, H-7), 1.40 (6H, s, H-10 and H-11); ^13^C-NMR (CDCl_3_, 125 MHz) δ 184.6 (C-6), 153.4 (C-8), 147.6 (C-5), 144.4 (C-2), 128.4 (C-3), 122.7 (C-7), 109.1 (C-4), 71.3 (C-9), 29.6 (C-10 and C-11); HREIMS *m*/*z* 180.0788 [M]^−^ (calcd. for C_10_H_12_O_3_, 180.0786).

### 4.4. HPLC-DAD Anlysis

Quantitative analysis was conducted using an Agilent 1200 series LC system. Data acquisition and processing were performed using the ChemStation software (version B.04.). The chromatographic separation of four compounds was performed at room temperature using an YMC-Triart C18 column (5 μm, 250 × 4.6 mm, YMC Co.) with a gradient solvent system of acetonitrile and water (45:55–55:45). The flow rate was maintained at 1 mL/min and the injection volume was 10 μL. Chromatograms were acquired at 254 nm for **1**–**3** and 210 nm for **4** using a DAD detector.

All the four compounds were weighed accurately, dissolved in methanol at 1.0 mg/mL, and diluted to yield a series of standard solutions at four different concentrations for quantitative analysis. The SC-CO_2_ extracts of PFCA and PFC were weighed accurately and dissolved in methanol at a concentration of 20 mg/mL. The standard and sample solutions were filtered through a syringe filter (0.45 μm) before HPLC analysis.

### 4.5. Measurement of NO Production on LPS-Stimulated RAW 264.7 Cells

NO formation was measured in cultured RAW 264.7 cells. RAW 264.7 cells were cultured in Dulbecco’s Modified Eagle’s Medium (DMEM) supplemented with 10% fetal bovine serum (FBS), penicillin (100 units/mL), and streptomycin (100 μg/mL) and incubated at 37 °C in 5% CO_2_ of humidified air. The cells were plated in a 96-well plate and then incubated for 24 h. The cells were pre-treated with various concentrations of compounds (6.25−50 μM) for 2 h, and then incubated in the medium with 1 μg/mL of LPS in the presence or absence of test samples for an additional 18 h. The media were collected and analyzed for nitrite accumulation by the Griess reaction. Briefly, 100 μL of Griess reagent—0.1% *N*-(1-naphthyl)ethylenediamine dihydrochloride in H_2_O and 1% sulfanilamide in 5% H_3_PO_4_—was added to 100 μL of each supernatant from LPS or sample-treated cells in 96-well plates. The absorbance was measured at 540 nm using an ELISA reader, and the nitrite concentration was determined by comparison with a sodium nitrite standard curve. The percentage inhibition was expressed as [1 − (NO level of test samples/NO level of vehicle-treated control)] × 100. The IC_50_ value, the sample concentration resulting in 50% inhibition of NO production, was determined by non-linear regression analysis (% inhibition versus concentration).

### 4.6. Cytotoxicity Assay

The EZ-Cytox cell viability assay kit was used to measure the cell viability. The cells were cultured in a 96-well plate at a density of 2 × 10^5^ cells/mL for 24 h. Compounds were dissolved in DMSO and incubated with the cells at the concentrations of 6.25, 12.5, 25, and 50 µM for an additional 24 h. After the incubation period, 10 μL solution of cell viability assay kit was added to each well and incubated for 4 h at 37 °C and 5% CO_2_. The index of cell viability was determined by measuring the formazan production using a spectrophotometer at an absorbance of 480 nm with a reference wavelength of 650 nm.

## 5. Conclusions

Our phytochemical investigation on the SC-CO_2_ extracts of the radiation mutant cultivar, PFCA, and the original cultivar, PFC, successfully led to the isolation of a new monoterpene, 9-hydroxy-isoegomaketone (**1**) and myricitrin (**4**), respectively, for the identification of unknown peaks of the HPLC chromatograms of PFCA and PFC. Compound **1** exhibited inhibitory activity on LPS-induced NO production in RAW 264.7 cells. Thus, **1** might be a potential standard for efficacious use of PFCA in inflammation disease, together with isoegomaketone (**2**), which is known as the major active standard in the SC-CO_2_ extract of PFCA. Our findings will be helpful for the quality control of PFCA to develop the medicine and dietary supplement related to anti-inflammation.

## Figures and Tables

**Table 1:** Linear range, regression equation, correlation coefficients, LODs, and LOQs for compounds.

Compound	*t*_R_ (min)	Linear Range (μg/mL)	Regression Equation (*y* = a*x* + b) ^1^	Correlation Coefficient (*r*^2^)	LOD ^2^ (μg/mL)	LOQ ^3^ (μg/mL)
**1**	5.78	20−100	*y* = 13.154*x* − 8.1524	0.9991	0.103	0.641
**2**	21.87	20−164	*y* = 44.574*x* − 27.463	0.9992	0.067	0.952
**3**	28.04	20−100	*y* = 7.8236*x* − 0.9952	0.9992	0.006	0.116
**4**	33.10	100−1000	*y* = 10.824*x* − 67.536	0.9992	0.124	0.375

^1^ *y* = peak area, *x* = concentration (μg/mL), a = slope, b = intercept; ^2^ LOD: 3.3 × (SD of the response/slope of the calibration curve); ^3^ LOQ: 10 × (SD of the response/slope of the calibration curve).

**Table 2:** The contents of compounds in the leaves of PFCA and PFC.

Peak	Compound	Contents (mg/g)	
		PFCA	PFC
1	9-hydroxy-isoegomaketone (**1**)	1.33 ± 0.07	0.15 ± 0.02
2	isoegomaketone (**2**)	2.76 ± 0.05	0.51 ± 0.02
3	perilla ketone (**3**)	6.96 ± 0.17	1.71 ± 0.12
4	myristicin (**4**)	0.042 ± 0.003	36.77 ± 5.60

## Supplementary Materials

Supplementary Materials are available online. Figure S1: ^1^H-NMR (500 MHz, CDCl_3_) spectrum of compound **1**, Figure S2: ^13^C-NMR (125 MHz, CDCl_3_) spectrum of compound **1**, Figure S3: ^1^H-^1^H COSY NMR spectrum of compound **1**, Figure S4: ^1^H-^13^C HMQC NMR spectrum of compound **1**, Figure S5: ^1^H-^13^C HMBC NMR spectrum of compound **1**.
