# Supplementary material for: A New Monoterpene from the Leaves of a Radiation Mutant Cultivar of Perilla frutescens var. crispa with Inhibitory Activity on LPS-Induced NO Production
Source: Molecules. 2017 Sep 4;22(9):1471. doi: 10.3390/molecules22091471 (PMC6151837; doi:10.3390/molecules22091471)
Supplement: Supplementary file 1 [file molecules-22-01471-s001.pdf]

Supplementary Materials: **A New Monoterpene from the Leaves of a Radiation Mutant Cultivar of *Perilla frutescens* var. *crispa* with Inhibitory Activity on LPS-Induced NO Production**

Bomi Nam, Yangkang So, Hyo-Young Kim, Jin-Baek Kim, Chang Hyun Jin and Ah-Reum Han *

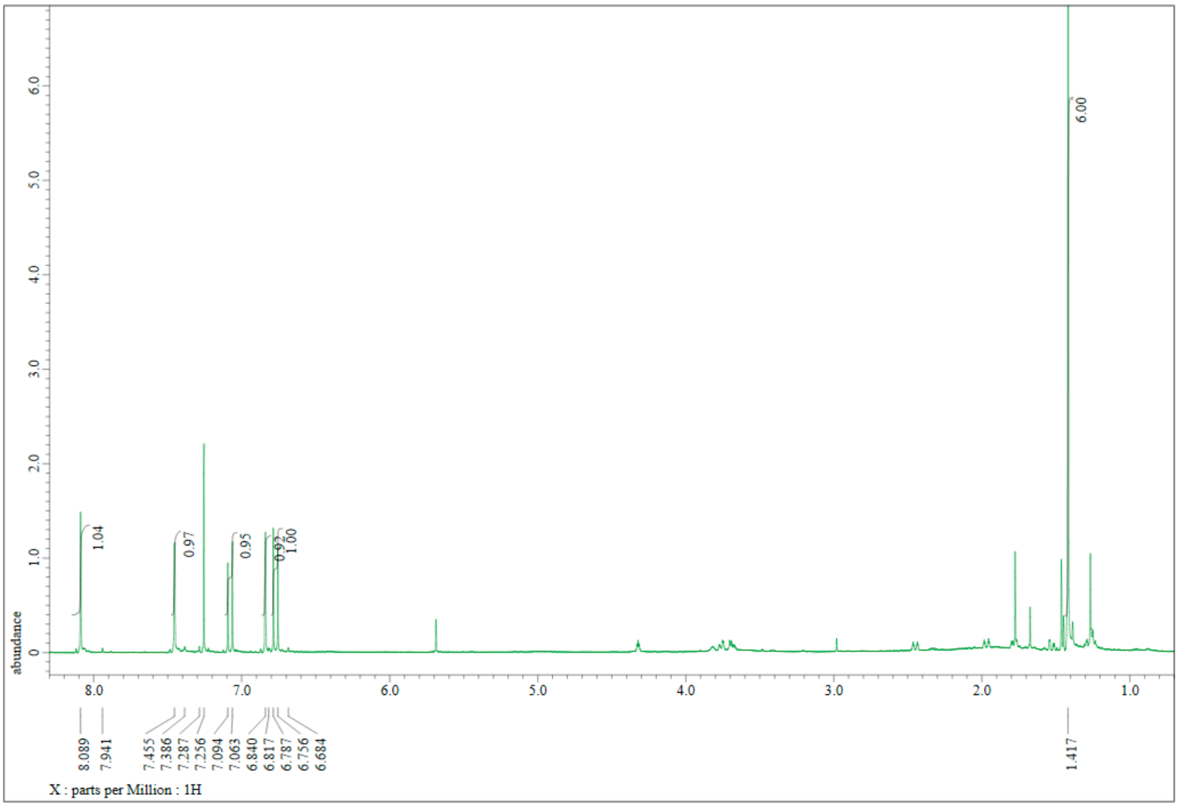

Figure S1. ^{1}H-NMR (500 MHz, $CDCl_3$) spectrum of compound **1**.

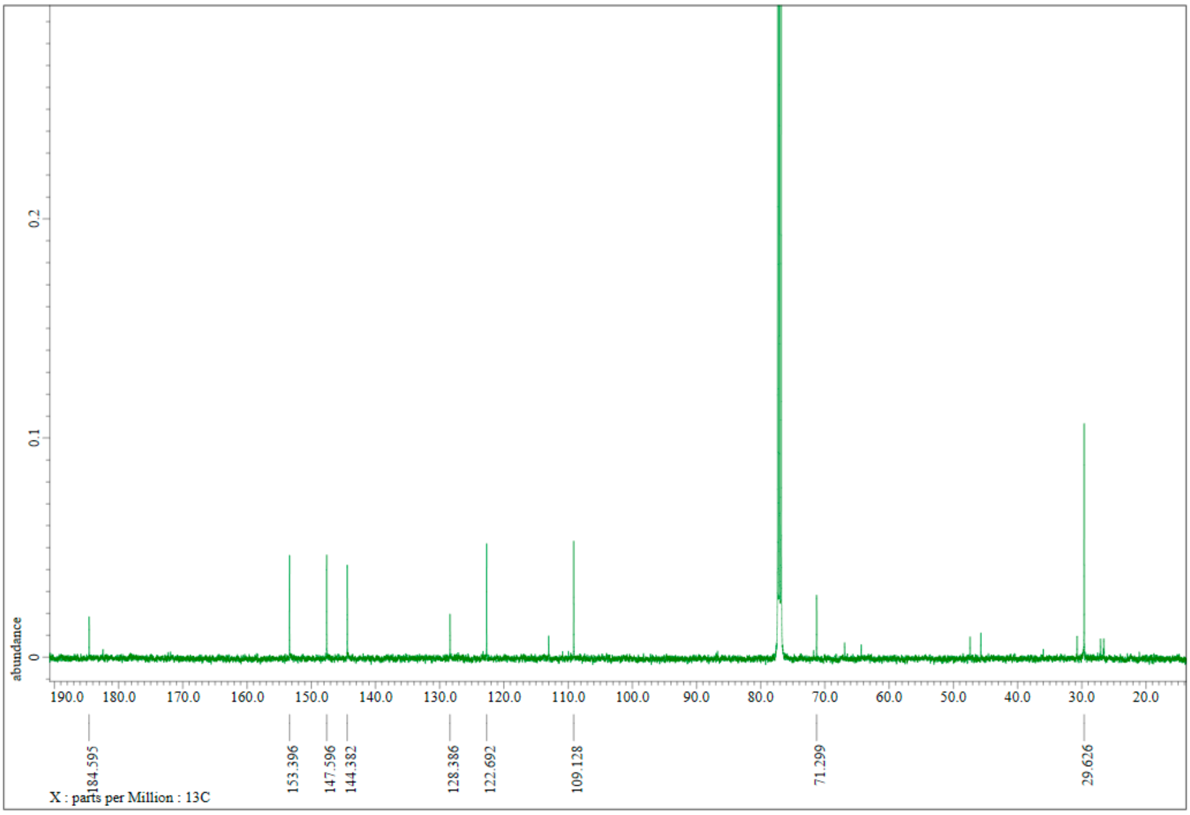

Figure S2. ^{13}C-NMR (125 MHz, $CDCl_3$) spectrum of compound **1**.

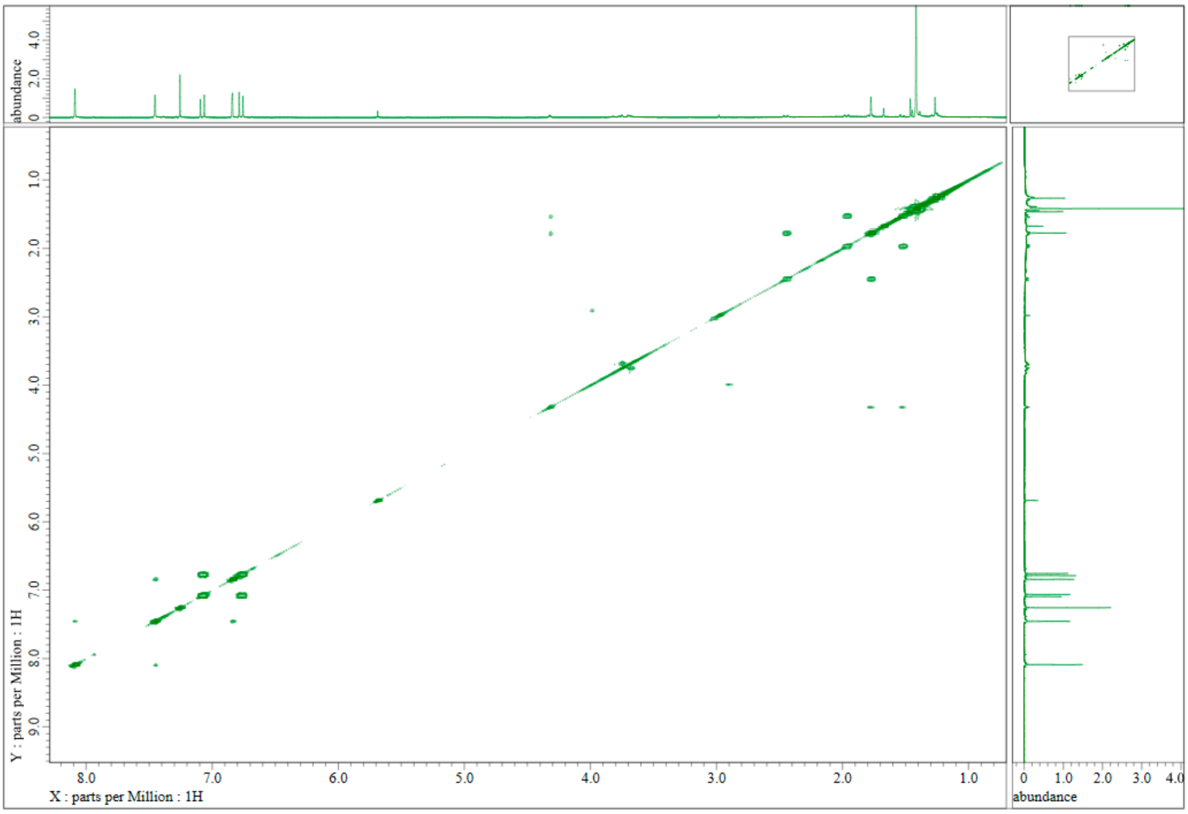

Figure S3. ^{1}H-^{1}H COSY NMR spectrum of compound **1**.

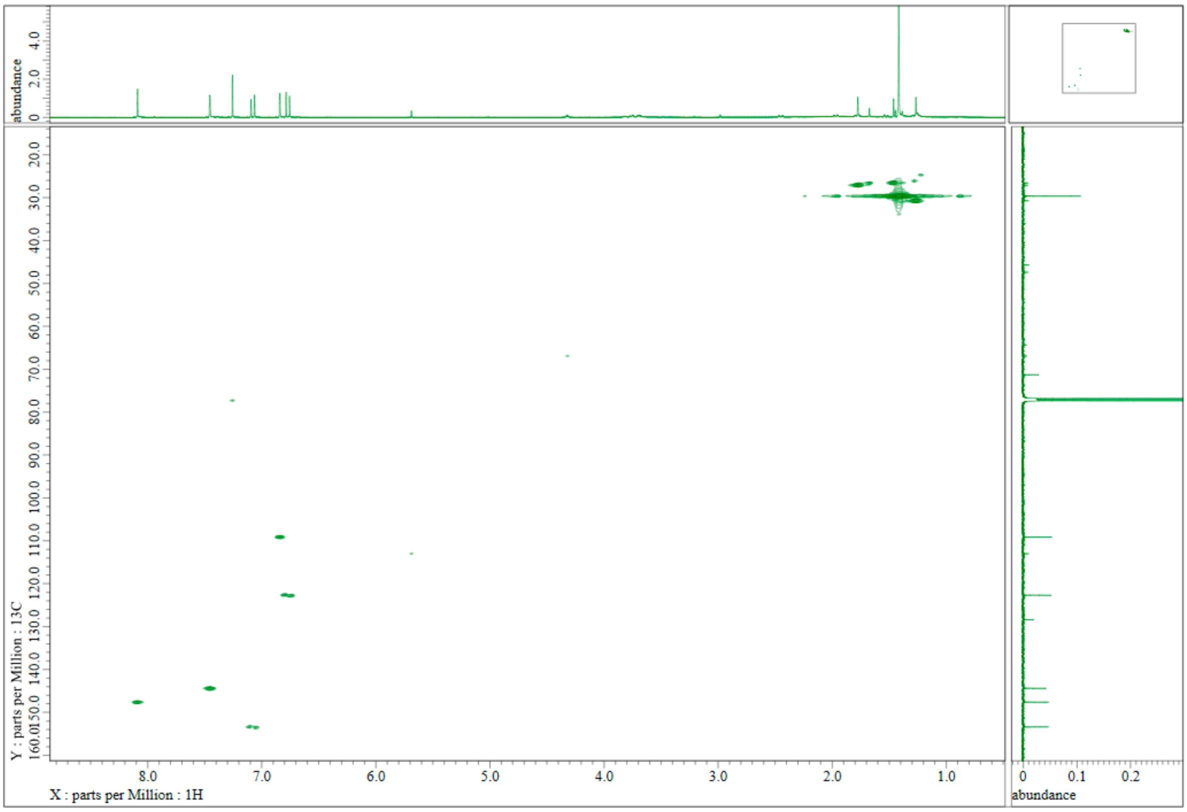

Figure S4. ^{1}H-^{13}C HMQC NMR spectrum of compound **1**.

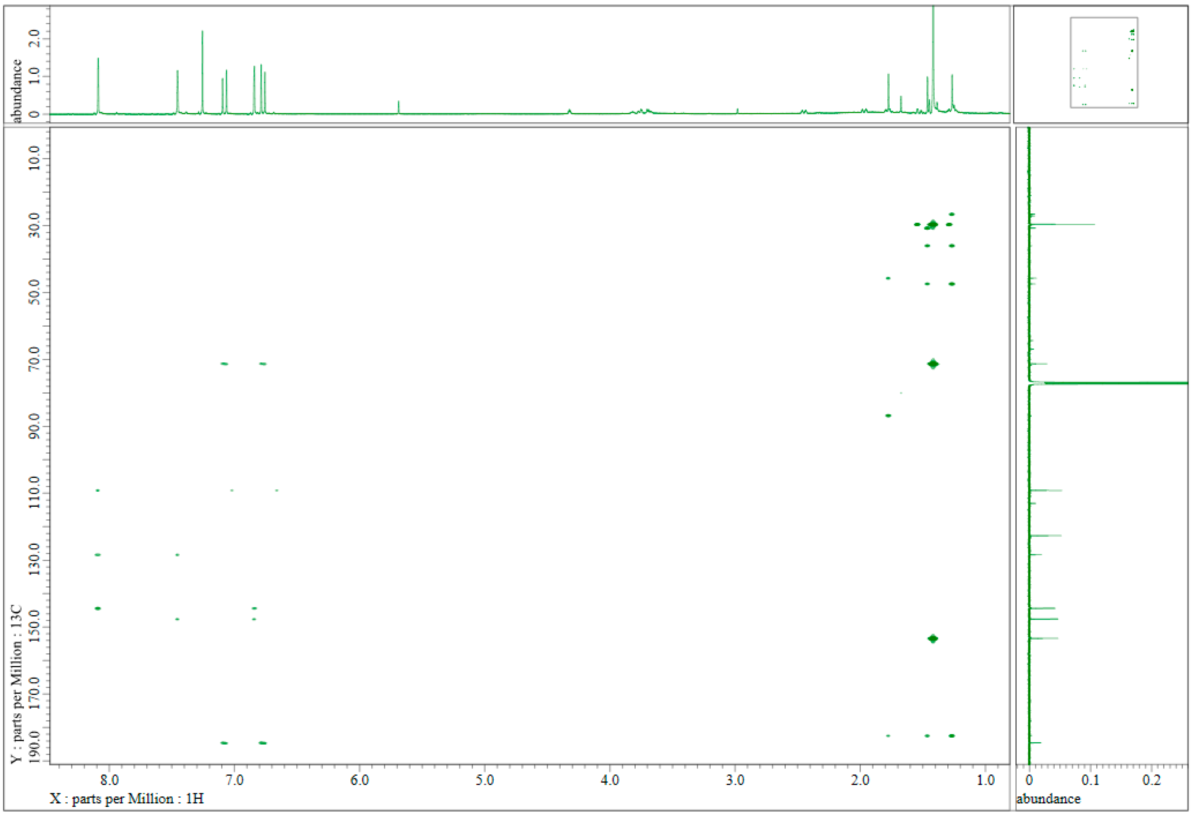

Figure S5. ^{1}H-^{13}C HMBC NMR spectrum of compound **1**.
